# Comparative Evaluation of the Effects of Fluoride Mouthrinse, Herbal Mouthrinse and Oil Pulling on the Caries Activity and *Streptococcus mutans* Count using Oratest and Dentocult SM Strip Mutans Kit

**DOI:** 10.5005/jp-journals-10005-1295

**Published:** 2015-08-11

**Authors:** Deepika Jauhari, Nikhil Srivastava, Vivek Rana, Preetika Chandna

**Affiliations:** Senior Lecturer, Department of Pedodontics and Preventive Dentistry, Jaipur Dental College, Jaipur, Rajasthan, India; Principal and Head, Department of Pedodontics and Preventive Dentistry Subharti Dental College, Meerut, Uttar Pradesh, India; Professor, Department of Pedodontics and Preventive Dentistry Subharti Dental College, Meerut, Uttar Pradesh, India; Reader. Department of Pedodontics and Preventive Dentistry Subharti Dental College, Meerut, Uttar Pradesh, India

**Keywords:** Caries activity, Dentocult SM strip mutans kit, Fluoride, Oil pulling, Oratest, Salvadora persica, Streptococcus mutans.

## Abstract

**Background:** As the technological level of healthcare increases, it is important not to lose sight of the basics of patient care. No matter how sophisticated dental techniques have become, preventive dentistry still remains the foundation for oral health. Therefore, antimicrobial mouthrinses are developed to provide an effective means of preventing colonization by micro-organisms.

**Aim:** The aim of this study was to evaluate and compare the antimicrobial activity of oil pulling, herbal mouthrinses and fluoride mouthwash on the caries activity and S. *mutans* counts in the saliva of children, using Oratest and Dentocult SM kit.

**Design:** Fifty-two healthy children between the age group of 6 to 12 years were selected for the study and divided into four groups based on the mouthrinse used as group 1: fluoride, group 2: herbal, group 3: oil pulling and group 4: control. The estimation of caries activity and *S. mutans* was done prior to and after the subjects were instructed to use the mouthrinse twice daily for a period of 2 weeks.

**Statistical analysis:** The comparisons were made by applying paired ‘t’ test with the level of significance set at p < 0.05. Difference between more than two mean values was done by using ANOVA and Post hoc Bonferroni test was used for multiple comparisons.

**Results and conclusion:** The efficacy of fluoride and herbal mouthrinses was found to be comparable while oil pulling did not provide any additional benefit to be used as an effective antimicrobial agent in reducing the bacterial colonization of an individual.

**How to cite this article:** Jauhari D, Srivastava N, Rana V, Chandna P. Comparative Evaluation of the Effects of Fluoride Mouthrinse, Herbal Mouthrinse and Oil Pulling on the Caries Activity and *Streptococcus mutans* Count using Oratest and Dentocult SM Strip Mutans Kit. Int J Clin Pediatr Dent 2015;8(2):114-118.

## INTRODUCTION

Good oral health is an integral component of good general health. Many children and adolescents have inadequate oral and general health because of active and uncontrolled dental caries.^1^ Oral micro-organisms are considered crucial for the initiation and progression of dental caries. Among the various micro-organisms studied, *S. mutans* have been regarded as one of the most virulent caries producing organisms.^2^

The tremendous backlog of unmet dental needs and ever increasing demand for care has made it quite obvious that dental diseases cannot be controlled by treatment alone. No matter how sophisticated dental techniques have become, preventive dentistry still remains the foundation on which oral healthcare must be built. Therefore, various chemotherapeutic agents have been developed for home use.^1^ Antimicrobial mouthrinses act as adjuncts to daily home care and provide an effective means of preventing colonization by micro-organisms. The use of dilute oral fluoride rinses and/or gels as an additional dental caries control measure has become another helpful adjunct. The cariopreventive action of fluoride has been due to its effect on teeth, bacteria and plaque.^3^ Fluoride alters the physiochemical properties of teeth by making them more resistant to acid dissolution due to the formation of fluorapatite.^4^ The upsurge in the prevalence of side effects of many synthetic medicines has encouraged scientists to research for plant based antimicrobial agents and the use of complementary and alternative medicine. Therefore, a growing number of consumers are embracing the philosophy that herbal based products are better for their health and the environment.^5^ Oil pulling has also been extensively used as a traditional Indian remedy to treat tooth decay, oral malodor, bleeding gums, dryness of throat and for strengthening the teeth, gums and jaw. The mechanism by which oil pulling causes reduction in *S. mutans* count may be attributed to the viscosity of oil which probably inhibits bacterial adhesion and plaque co-aggregation. Various oils, like refined sunflower oil, sesame oil, olive oil can be used for the oil pulling.

An increase in the prevalence of dental caries among young children has advocated the use of number of caries activity tests. Oratest is simple noninvasive method to estimate oral microbial levels, based on the rate of oxygen depletion in expectorated milk samples. Dentocult SM strip mutans kit is another simple and reliable method of chairside evaluation of *S. mutans* assuring greater patient compliance especially for young subjects and needs minimal armamentarium.

There is clinically relevant evidence to suggest that mouthrinses containing active agents are effective against *S. mutans* but limited information is available regarding the comparative effects of these active agents (Sodium fluoride mouthrinse, herbal mouthrinses and oil pulling) on caries activity and *S. mutans* using oratest and dentocult SM strip mutans kit. The aim of this study was to compare the antimicrobial efficacy of fluoride mouthrinse, herbal mouthrinse and oil pulling on the caries activity and *S. mutans* count using oratest and dentocult SM strip mutans kit respectively.

## MATERIALS AND METHODS

The study group in the present study consisted of 52 subjects in the age of 6 to 12 years. The study design selected was randomized double blinded controlled trial. Ethical clearance to conduct the study was obtained from the Institutional Ethical Committee (IEC).

Inclusion criteria involved good general health of the children and agreement to comply with the study visits and procedures. However, patients with the history of antibiotic use in the past 3 months and any fluoride treatment in the past 2 weeks were excluded from the study.

The children personal details, past medical history, past dental history, frequency of brushing, sweets/snacks intake were taken from the parents. Once the entire procedure was explained to the parents of the children included in the study, written consent was obtained. All 52 children were randomly divided in four groups of 13 children each as follows:

- *Group 1:* Fluoride mouthrinse containing 200 ppm sodium fluoride.
- *Group 2:* Herbal mouthrinse containing active ingredient as Salvadora Persica (5 mg).
- *Group 3:* Oil pulling.
- *Group 4:* Distilled water (control).

The subjects were instructed to use the mouthrinses and oil pulling technique twice daily for a period of 2 weeks. Caries activity and S. mutans counts were estimated using oratest and dentocult SM Strip mutans Kit prior to the use of mouthrinses and oil pulling technique and repeated again after a period of 2 weeks.

### Oratest Procedure

All children were asked to rinse their mouth vigorously for 30 seconds with 10 ml of sterilized milk. The expectorate was collected in a sterile beaker and 3 ml of expectorate was transferred with a disposable syringe to a screw cap test tube containing 0.12 ml of 0.1% methylene blue (prepared by mixing 100 mg of methylene blue in 100 ml of distilled water). The expectorated milk and methylene blue was thoroughly mixed and a mirror was used to detect any color change (blue to white) at the bottom of the tube every 5 minutes. The time taken for the initiation of color change was recorded.

### Dentocult SM Strip Mutans Procedure

**Fig. 1:**
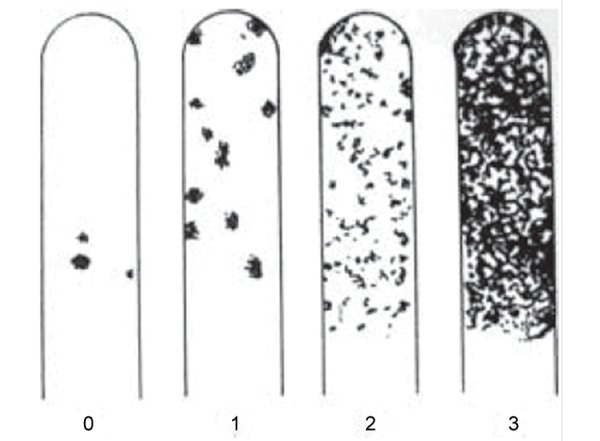
Assessment of S. *mutans* growth on the strips of Dentocult SM Strip mutans kit

A paraffin tablet was given to each subject to chew for 1 minute. Excess saliva was swallowed. The rough surface of dentocult SM saliva strip was pressed against the saliva on the tongue and the strip was removed through gently closed lips. The strips were placed in the selective culture broth with the smooth surface clipped and attached to the cap. The vials were incubated in an upright position at 37°C for 48 hours with the cap opened one quarter to allow growth of microorganisms. The growth on the strips was assessed according to the manufacturer’s chart (Fig. 1).

Class 0―< 10,000 CFU/ml, Class 1―< 1,00,000 CFU/ml, Class 2―1,00,000-10,00,000 CFU/ml, Class 3―> 10,00,000 CFU/ml

All the data obtained were calculated and statistically analyzed. Values were expressed in the form of mean, standard deviation and standard variation of mean. The comparisons were made by applying paired ‘t’ test with the level of significance set at p < 0.05. Difference between more than 2 mean values was done by using analysis of variance (ANOVA) test and post hoc Bonferroni test was used for multiple comparisons after the application of the ANOVA test for comparison within the groups.

## RESULTS

At base line, when the colony forming units were recorded using Dentocult SM strip mutans kit, all the groups did not differ significantly from each other with respect to the total number of mutans streptococci CFU/ml of saliva. After 2 weeks of the treatment, there was significant decrease in mutans streptococci count (CFU/ml of saliva) in the groups 1 and 2 (Table 1).

**Graph 1:**
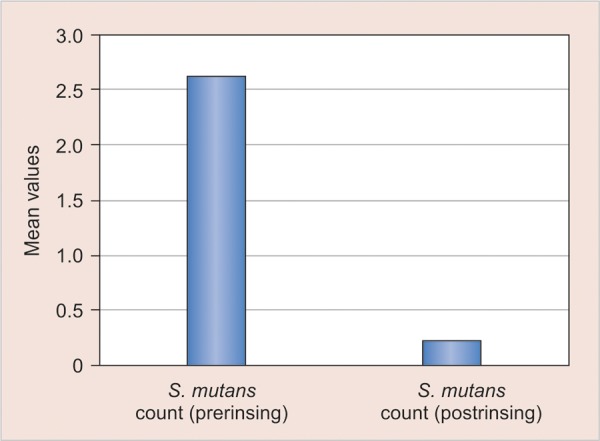
Mean comparison of pre and post-treatment values of S. *mutans* count in group 1 (fluoride mouthrinse)

**Graph 2:**
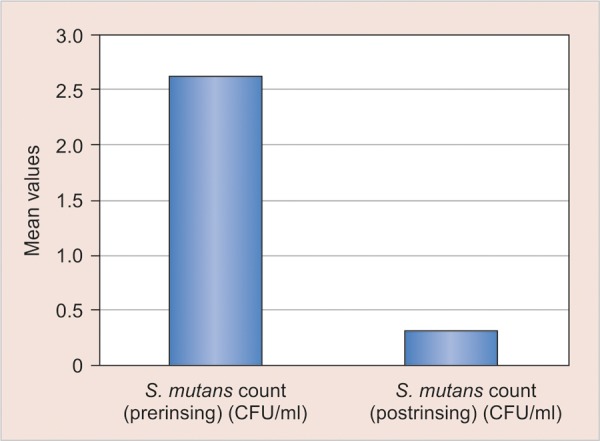
Mean comparison of pre and post-treatment values of S. *mutans* count in group 2 (herbal mouthrinse)

**Graph 3:**
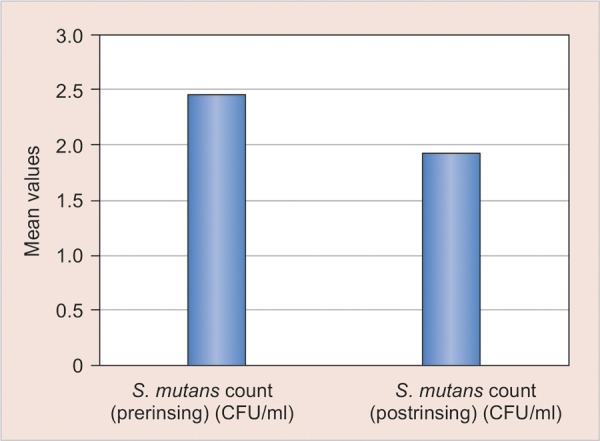
Mean comparison of pre and post-treatment values of S. *mutans* count in group 3 (oil pulling)

No statistical significant difference was seen among all groups when the intergroup comparison were made at the baseline (Table 2) however after 2 weeks of treatment schedule maximum reduction in the *S. Mutans* was seen in the fluoride and herbal mouthrinse group (Table 3) (Graphs 1 and 2) while there was no statistical significant difference in the oil pulling group (Graph 3).

**Table 1:** **Table 1:** Comparison of pre- and post-treatment mean values of S. *mutans* count in the study and control groups using the paired ‘t’ test

Groups		*Mean S. mutans count prerinsing*		*Mean S. mutans count prerinsing*		Mean standard deviation		p value	
1		2.62		0.23		0.650		0.000	
2		2.61		0.31		0.751		0.000	
		2.46		1.92		0.967		0.068	
4		2.38		1.97		0.796		0.078	

**Graph 4:**
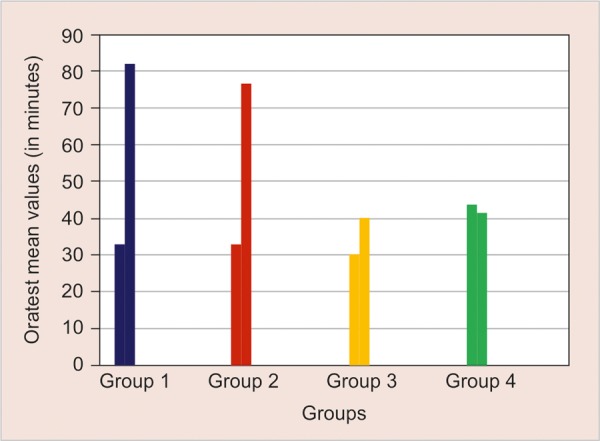
Comparative evaluation of the prerinsing and postrinsing Oratest counts in minutes in the various mouth rinses group

The mean values of the prerinsing and postrinsing Oratest mean values (in minutes) of the control and the study group were also calculated and tabulated Table 4 (Graph 4). Significant correlation was found with the use of mouthrinse and time required for color change.

**Table 2:** **Table 2:** Intergroup comparison between the mean difference of the prerinsing S. *Mutans* counts between the control and study groups.

Groups		Groups		*Mean difference*		p-value*	
Group 1 (Fluoride mouthrinse)		2		0.000		1.000	
		3		0.154		1.000	
		4		0.231		1.000	
Group 2		1		0.000		1.000	
		3		0.154		1.000	
		4		0.231		1.000	
Group 3 (Oil pulling)		1		-0.154		1.000	
		2		-0.154		1.000	
		4		0.077		1.000	
Group 4 (Control group)		1		-0.231		1.000	
		2		-0.231		1.000	
		4		-0.077		1.000	

**Table 3:** **Table 3:** Intergroup comparison between the mean difference of the postrinsing *S. mutans* counts between the control and study groups

*Groups*		*Groups*		*Mean difference*		p-value*	
Group 1 (Fluoride mouthrinse)		Herbal		-0.077		1.000	
		mouthrinse		-1.692		0.000	
		Oil pulling		-2.154		0.000	
		Control					
Group 2 (Herbal mouthrinse)		Fluoride		0.077		1.000	
		mouthrinse		-1.615		0.000	
		Oil pulling		-2.077		0.000	
		Control					
Group 3 (Oil pulling)		Fluoride		1.692		0.000	
		mouthrinse		1.615		0.000	
		Herbal		-0.462		0.743	
		mouthrinse					
		Control					
Group 4 (Control group)		Fluoride		2.154		0.000	
		mouthrinse		2.077		0.000	
		Herbal				0.743	
		mouthrinse					
		Oil pulling		0.462			

**Table 4:** **Table 4:** Intergroup comparison of the mean values of the pre rinsing and postrinsing oratest counts (in minutes) between the study and control groups

*Groups*		*Mean values of the pre-rinsing and postrinsing oratest counts*		*Standard deviation*		p-value*	
1		33.08		6.63		0.000	
		81.92		18.09			
2		32.69		10.73		0.000	
		76.92		19.64			
3		30.38		11.27		0.097	
		40.38		15.61			
4		43.85		21.62		0.291	
		41.54		22.77			

## DISCUSSION

The present study was a comparison of antimicrobial efficacy of fluoride mouthrinse, herbal mouthrinse containing salvadora persica and oil pulling technique using Sesame oil on the caries activity and *S. mutans* counts using oratest and dentocult SM strip mutans kit. Each of the active agents has a different mechanism of action. Fluoride alters the physiochemical properties of teeth by making them more resistant to acid dissolution due to the formation of fluorapatite. It also increases the post eruptive maturation, enhances remineralization and inhibits demineralization, inhibits various enzymes, like enolases and, therefore, the transport of glucose is inhibited.^4^

Salvadora Persica extracts exerts antimicrobial effects on *S. mutans* due to the interaction with bacteria, which prevents their attachment on the tooth surface.^5^

Sesame oil has sesamin, sesamolin and sesaminol which has action on the living tissues, like the detoxification of toxins, antioxidant effect, potentiation of action of vitamin E, prevention of lipid peroxidation and antibiotic effect.

In the present study, when the *S. mutans* count was assessed using Dentocult SM strip mutans kit, marked reduction of the bacterial counts was observed in the subjects after 2 weeks of treatment using fluoride mouthrinse. The results of the present study are in accordance with a study done by Kulkarni VV6 where there was significant decrease in *S. mutans* count in study groups as compared to the control group after 2 weeks of treatment of rinsing with sodium fluoride, chlorhexidine and triclosan mouthrinse. Similar results were also obtained in the study done by Jeevarathan J^7^ and Aminabadi NA^8^ who concluded that the weekly use of 0.2% sodium fluoride mouthwash program could play an important role in the improvement of oral and dental health among children of school age. Ripa LW^9^ conducted a 2 years study to evaluate the effect of 0.2% neutral sodium fluoride mouthrinsing and showed that difference in caries prevalence was seen in children who participated in the rinsing program for 2 years compared with baseline caries scores of children who never rinsed.

The results of the study also showed that use of Herbal mouthrinse (containing 5 mg of Salvadora Persica) resulted in significant reduction in the bacterial counts post rinsing and the results were comparable to the subjects using fluoride mouthrinse. It has been demonstrated that chemical compounds, such as sodium chloride, calcium oxalate, silica, fluoride, sulfated compounds, Saponin, Flavonoid, an alkaloid Salvadorin, Trim Ethylamine and Benzyl Isothiocyanate vitamin C and tannic acid have been found in Salvadora persica plant. Calcium salts and fluoride are quite effective in preventing dental caries.^11^ The results are in accordance to the results obtained by Bhatt PK^10^ and Salehi P^11^ who showed that Salvadora persica extract showed significant reduction of microbial count of *S. mutans.*

The efficacy of oil pulling as an antimicrobial agent has been demonstrated in the previous studies conducted by Anand TD^12^, Thaweboon S^13^ and Asokan S^14^. However, no significant reduction in the bacterial counts was observed in the present study on the mean comparison of the pre rinsing and post rinsing *S. mutans* colony counts.

On intergroup comparison of the mean difference of the pre and post rinsing *S. mutans* counts, fluoride and herbal mouthrinses showed highest but equal efficacy in reducing the *S. mutans* count.

The efficacy of Oratest as a simple chair side caries activity test was also evaluated in the present study and it was seen that greater was the amount of bacterial colonization, lesser was the amount of time taken for the change in the color of methylene blue and vice versa, and thus it was inferred that oratest provided a reliable estimate of the caries activity. The results of the present study are in accordance to the study done by Bhasin S^15^ where maximum time taken for color change [91 minutes ± 21] was observed in children with 1 to 5 carious teeth and the minimum time taken for the color change (28 minutes ± 7) was in children with 11 to 15 carious lesions.

It has been hypothesized that Oratest is based on the rate of oxygen depletion by micro-organisms. Under aerobic conditions the bacterial enzyme; aerobic dehy-drogenase transfers electrons or protons to oxygen. Once oxygen gets utilized by the aerobic organisms and an anaerobic environment is attained, methylene blue (redox indicator) acts as an electron acceptor and gets reduced to leukomethylene blue. The metabolic activity of the aerobic microorganism is reflected by the reduction of methylene blue to leukomethylene blue.^15^

## CONCLUSION

On comparison of the antimicrobial efficacy of fluoride mouthrinse, herbal mouthrinse and oil pulling on the caries activity and *S. mutans* counts, fluoride and herbal mouthrinses were equally effective in reducing the caries activity and showed a marked reduction in *S. mutans* count. However, oil pulling did not show promising results as an effective antimicrobial agent in reducing bacterial colonization.

Further studies must be conducted using a larger sample size and long-term follow-up to evaluate and compare the antimicrobial activity of oil pulling, herbal and fluoride mouthrinses on the caries activity and *S. mutans* counts in the saliva of children, using Oratest and Dentocult SM kit.
